# Influence of Psychosocial Variables on the Health of People Living in Housing Exclusion

**DOI:** 10.3390/ijerph17238983

**Published:** 2020-12-02

**Authors:** Fernando Fajardo-Bullón, Jesús Pérez-Mayo, Igor Esnaola, Isobel Anderson, Marcus Knutagård

**Affiliations:** 1 Department of Psychology, Faculty of Education and Psychology, University of Extremadura, Avenida de Elvas s/n, 06006 Badajoz, Spain; 2 Department of Economics, University of Extremadura, 06006 Badajoz, Spain; jperez@unex.es; 3 Department of Development and Educational Psychology, Faculty of Education, University of the Basque Country, UPV/EHU, Avenida de Tolosa, 70, San Sebastián, 20018 Leioa, Spain; 4 Faculty of Social Sciences, University of Stirling, Stirling FK9 4LA, UK; isobel.anderson@stir.ac.uk; 5 School of Social Work, Lund University, Box 23, 221 00 Lund, Sweden; marcus.knutagard@soch.lu.se

**Keywords:** housing exclusion, self-rated health, psychosocial variables, Spain

## Abstract

The aim of this study was to analyze the influence of some personal characteristics, health variables, and social support on the self-rated health of people in housing exclusion in Spain. For that purpose, we used the FOESSA Survey of Social Integration and Needs database, with a final sample of 1574 households. Being more educated and reporting a good life satisfaction stood out as the main factors preventing worse health status. Furthermore, results showed that being female, experiencing poverty-related food insecurity, not having health insurance, experiencing widowhood or partner bereavement, and having caring responsibilities for others or having a disabled person in the household are associated with increased reporting of regular or poor health. On the other hand, being young, having a diagnosed/long-term illness, and a big household size are preventive factors for good health. These results allowed identifying risk and prevention factors to inform interventions to improve the health of those living in housing exclusion. Promoting better education levels, social support, and overall life satisfaction could be important to improve health in this population. Developing social support policies for caring responsibilities and food insecurity must be a priority to improve the health of people living in housing exclusion.

## 1. Introduction

While homelessness is an extreme situation of social exclusion, other dimensions of housing exclusion and the residential environment also influence individual and public health. This paper draws on the widely used European Typology of Homelessness and Housing Exclusion (ETHOS) framework [1] and the 2019 FOESSA (Fomento de Estudios Sociales y Sociología Aplicada in Spanish) survey of social exclusion in Spain [2] to examine the influence of key psychosocial variables on the health of people living in situations of housing exclusion. Developed for the European Federation of Organizations Working with the people who are homeless (FEANTSA) [1], the ETHOS framework establishes four main concepts of housing exclusion: rooflessness, houselessness, insecure housing, and inadequate housing. The roofless and houseless dimensions together define homelessness; insecure and inadequate accommodation refer to housing exclusion [1,3,4]. The European consensus conference on homelessness in 2010 and the growing attention at the European level to strengthen the fight against homelessness helped shape the creation of the first Spanish “Comprehensive National Homelessness Strategy, 2015–2020” (ENI-PSH in Spanish), adopted in November 2015 [5]. Robust analysis of the variables that influence the status and risks of homeless people is key to achieving the objectives of national and European strategies against poverty, social exclusion, and inequality [6].

The most recent national public survey of homelessness in Spain was in 2012 [7]; however, ENI-PSH estimated that the total number of homeless people in Spain (without a house or a roof) was around 33,000 in 2014 [5]. However, the Spanish homelessness nongovernmental organization (NGO), Caritas, estimated that they were accompanying and caring for approximately 40,000 homeless people in Spain in 2019 [8]. Moreover, the independent FOESSA foundation conducted a national survey of social exclusion in Spain in 2018. The survey results showed that 800,000 households in Spain were experiencing housing insecurity (four in 100 households), 1,300,000 households were living in inadequate housing (seven in 100 households), and 150,000 households were facing both housing insecurity and inadequate housing at the same time [2]. Other evidence from Spain indicates that between January and August 2019, some 100 evictions a day were estimated to have taken place due to nonpayment of rent and 42 per day due to nonpayment of a mortgage loan [9]. These figures indicate significant housing stress resulting from a complex range of factors whereby housing insecurity carries important implications for systematic responses to homelessness [10]. Support to avoid eviction where possible or to find accommodation, along with integrated case management to support complex needs, is widely held as the key solution for the prevention of homelessness [11]. Internationally, numerous evidence-based strategies are being employed to end homelessness by increasing access to housing options and supportive services for housing stability [12,13]. At the same time, housing is an important determinant of health, and substandard housing is a major public health issue [14,15]. Using the ETHOS classification [1], Fajardo-Bullón et al. (2019) [14] analyzed the variables that influence the self-rated heath (SRH) of homeless people in Spain, but there has been no prior analysis of influences on the health of the population living in housing exclusion in the FOESSA Spanish study.

The quality of housing conditions and the housing environment are recognized internationally as key settings that affect human health [16,17]. The National Housing Federation of the United Kingdom (UK) spends around GBP 2.5 billion per annum addressing housing and health related conditions across the UK [18], and the total direct and indirect costs of the impact of inadequate housing on health in Spain exceed EUR 22,350 million per year, one of the highest amounts in the entire European Union (EU)-28 [19]. Homelessness and housing exclusion are rightly also receiving more attention at the EU level [20]. A main driver for the continuing effort is the stark reality that more and more people are unable to secure and keep a decent home. According to the Fourth Overview of Housing Exclusion in Europe [21], in 2017, the proportion of Spanish households overburdened by housing costs was 36.5% of the population in poverty (38% average in the EU-28) and 9.8% (10.4% average in the EU-28) of the total population. Rodriguez Cabrero et al. (2019) declared that “the share of households with rent or mortgage arrears was the seventh highest in Europe: 10.5% among poor households and 3.8% among the total population (well above the EU average). Some 19.4% of poor households experienced financial difficulty” (p. 6) [8]. Existing data predate the 2020 coronavirus pandemic, and the ensuing economic crisis will almost certainly increase pressure with respect to housing systems and housing exclusion [22]. The World Health Organization has a very clear target: to assess and quantify the effect on health of housing conditions and how housing risks contribute to environmental and health inequalities [23]. In Spain, 5% of the Spanish population lives in insecure housing and 10% in inadequate housing, but we do not know how this affects the health of the population. While there is some existing evidence on how living in insecure and inadequate dwellings affects health, there is no rigorous data on the specific risks and preventive factors that influence the SRH of people experiencing housing exclusion [18].

### 1.1. Self-Rated Health (SRH)

Self-rated health (SRH), also called self-assessed health or self-perceived health, is one of the most commonly analyzed variables in epidemiology but remains relatively rarely used in studies of health and housing. SRH is based on individuals evaluating their own health status on a four- or five-point scale, which can then be used to analyze risk factors associated with individual health status [24]. In some studies of the relationship between SRH and physical health within the general population, no association was found between SRH and health factors such as hospital morbidity, occupational accidents, and consumption of medicines among the Spanish general population [25]. However, more recent studies in the general Spanish population demonstrated the relationship between objective health and SRH [26,27], whereas studies in other countries identified relationships with physical disability [28], functional performance, physical and social activity [29], or mental health [30]. The use of SRH measures in national and international surveys has increased following demonstration of the relationship between SRH and mortality [31], and SRH has become an established tool in national surveys [32] and for measuring health characteristics and inequalities [24]. Given its established reliability [33], the SRH measure is a helpful tool for predicting future health issues, as well as measuring current health [34,35]. There is some evidence that SRH is a more effective predictor of mortality among men than women and that there are also some differences in effectiveness across ethnic and socio economic groups [36]. Likewise, Zajacova and Woo (2016) found SRH to be less reliable among older populations, possibly due to variations in self-evaluation over the life course and requiring care in comparing different age groups [37]. Despite these limitations, SRH remains a valuable indicator of health-related quality of life (HRQoL), which conveys individuals’ own evaluation of their physical, mental, and social health status, drawing on their perceptions and experiences [14,38,39].

### 1.2. Life Satisfaction

Life satisfaction (LS) can be defined as an individual’s overall appraisal of the quality of their life [40]. It is the ultimate goal of human development and it is important to subjective wellbeing (SWB) and adaptive psychosocial functioning [41] as a positive indicator of psychological wellbeing [42].

### 1.3. Social Support

Social support as a multidimensional construct encompassing the type of interpersonal interaction and relationship experienced by an individual, in addition to a belief that they are cared for and loved, esteemed, and valued, and that they are a part of the communication network [43,44,45]. The two main aspects of received and perceived social support are considered in current literature; while received social support implies the particular supportive behavior which is provided to recipients by their supportive networks, perceived social support, as a subjective part of this concept, refers to the recipient’s perceptions regarding how existing support is made available to satisfy their needs [46,47].

### 1.4. Personal Variables and SRH

Gender is a key variable where higher mortality rates are reported for males than females, even though women tend to report more symptoms and use of health services, as well as poorer SRH, than men [48]. However, a study of the Spanish working population by Pinillos-Franco and García-Prieto [49] found that the gap in self-rated health is related to education levels, with women’s health being poorer among the less educated, reflecting their precarious labor-market and household conditions. Zajacova, Huzunbazar, and Todd [50] also found that the tendency of women to register poorer SRH was lower for older age groups, and other studies have indicated that women’s health ratings may be lower than men’s during early adulthood [51]. However, evidence also suggests that this gender difference may reduce with age [52]. To sum up the evidence to date, men and women appear to evaluate their health differently [53] and SRH tends to predict mortality better for men than women [33,35]. However, Zajacova et al. (2017) [50] found that men and women’s concurrent SRH validity was similar for comparable health factors.

As health problems increase with age, older people are more likely to report poorer SRH [49,50]. For the Spanish population, previous research has indicated that being female and aged over 50 are characteristics associated with a higher probability of poor health [14] and being homeless [54]. Having a lower level of education is also a health risk variable in the homeless population living in shelters [55]. Life satisfaction is positively linked to longevity, social relationships, health, SRH, and positive health behaviors and inversely related to psychiatric morbidity, and all-cause, disease-specific, and injury mortality [56,57,58,59]. Overall, evidence suggests a positive relationship between SRH and life satisfaction [60,61,62]. Evidence to date has not, however, examined the influence of the characteristics of people living in housing exclusion on SRH in Spain since the 2008 economic crisis. The paper seeks to fill this gap by addressing the question of whether some sociodemographic variables such as gender, age, level of education, and life satisfaction influence the health of people in the housing exclusion situation in Spain.

### 1.5. Health Variables and SRH

This study was conducted in the aftermath of the Great Recession where the financial burden of housing costs in Spain increased, as did the number of evictions. A further economic recession is expected in Spain in the coming years, following the impact of the 2020 coronavirus pandemic. The expectation in the European Union is that the unemployed population in Spain will increase to 20% in 2021. In light of this problematic situation, it is important to know what health variables can reduce the impact on SRH and what other risk factors could increase it, in order to help prevent poor health and optimize positive health outcomes [63]. It is, therefore, important to consider if having a diagnosed long-term illness could be a risk factor for SRH [64] or could be a relevant factor to be aware of in order to acknowledge the importance of taking care of the health of people living in housing exclusion.

In a recent study of the Spanish homeless population living in shelters, having a disability and not having a security card were risk factors for SRH [14]; however, no information was provided about how they can affect the Spanish population under specific housing exclusion. In the same direction, it was demonstrated that having a health card in Spain was a preventative factor for homeless people living in shelters and on the street. As Spain has a public health system, this analysis examines whether being uninsured affects the health of Spanish people in housing exclusion.

From 2013 to 2019, the price of rent in Spain increased by 50%, and 64% of low-income families spent more than 40% of their income paying rent [65]. Consequently, many households facing social exclusion had to be helped by NGOs providing free food, raising the question of whether experiencing poverty-related hunger or food insecurity influences the health of Spanish people in housing exclusion. There are two categories of indicators of food insecurity. The first is based on the adequacy of food consumption, and the second is based on the severity of constrained food access [66]. In this study, the second category was used.

### 1.6. Social Support and SRH

The influence of social support on individuals’ disease recovery, coping resources, and HRQoL is notable [67,68,69,70,71]. Several studies demonstrated that, in both Western and Eastern communities, perceived social support is positively associated with HRQoL in certain groups, e.g., those with acute or chronic diseases [72,73,74,75,76,77,78,79], the elderly [80,81], and immigrant workers and employees [82,83]. Across studies to date, higher scores on affective, confidant, and instrumental support correlated with higher physical and mental health scores. Strong, positive correlations have also been found between perceived social support and self-rated physical and mental health [84].

The remainder of this paper presents the research method for and findings from an analysis of the influence of personal characteristics (gender, age, education level, and overall life satisfaction), health variables (diagnosed long-term illness, experiencing poverty related hunger, and being uninsured), and social support (disabled person in household, divorced/relationship breakdown, giving care, widow/bereavement, and household size) on the SRH of people in housing exclusion in Spain.

### 1.7. The Present Study

As noted above, even though Fajardo-Bullón et al. (2019) [14] analyzed the variables that influence the self-rated heath (SRH) of homeless people in Spain, there has been no prior analysis of influences on the health of the population living in housing exclusion in a Spanish sample. Therefore, this paper presents new analysis, and the aim of the study was threefold: (1) to analyze the influence of some personal characteristics (gender, age, education level, and overall life satisfaction) on the SRH of people experiencing housing exclusion in Spain, (2) to analyze the influence of some health variables (diagnosed long term illness, experiencing poverty related hunger, and being uninsured) on the SRH of people experiencing housing exclusion in Spain, and (3) to analyze the influence of social support (e.g., with a disability or socioeconomic household vulnerability) on the SRH of people experiencing housing exclusion in Spain. By identifying risk and prevention factors, the results aim to inform interventions to improve the health of those living in housing exclusion in Spain. For these purposes, a logistic regression model was used in order to incorporate all the variables in a unique and validated model. More information about the variables is presented in Section 2.

## 2. Materials and Methods

The estimation process and analysis performed in this paper used the 2018 EINSFOESSA (FOESSA Survey of Social Integration and Needs) database. This survey was collected by the independent, nonprofit Spanish foundation, FOESSA, for the FOESSA Report on Exclusion and Social Development in Spain [2], a widely recognized and highly regarded evidence base on social exclusion in Spain. The core study was conducted by more than 125 researchers from 30 universities and 15 social institutions and NGOs. It is one of the largest surveys funded and carried out by an independent institution in Spain and is accepted as fully comparable with the official statistics, including the EU Survey on Income and Living Conditions (EU-SILC). FOESSA allows any researcher to use the microdata upon request, so that secondary analyses can be conducted with this information. This survey was selected instead of other official surveys because of the detailed information about housing, which allows the analysis of housing exclusion by applying the ETHOS classification. A particular strength of the EINSFOESSA survey is that it includes categories of housing exclusion such as illegal occupation, nonconventional dwellings, or temporary structures, which are usually excluded from analysis in official databases such as EU-SILC or the National Health Survey, which are based on dwellings counted in the official census.

The EINSFOESSA survey was carried out by means of personal interviews using a structured questionnaire, which was precoded in 99% of the variables. Interviews were conducted between 19 January and 23 April 2018. A two-stage geographical selection procedure was used, with a first selection of census sections and a second selection using random routes. The pretrained interviewers worked on a total of 716 routes distributed in 464 municipalities in Spain. The study was performed in accordance with the Declaration of Helsinki, assuring anonymity in the answers, the confidentiality of the obtained data, and its exclusive use for research purposes [2].

The household was the unit of analysis of the survey, with 11,615 households sampled and a sampling error of ±5%, giving a sample size almost equal to the European Union Statistics on Income and Living Conditions (EU-SILC) sample for Spain. For our secondary analysis of people experiencing housing exclusion, a subsample of households was extracted from the dataset. The EINSFOESSA survey utilizes two key definitions of housing exclusion which are very close to the ETHOS classification [2], giving a final sample of 1574 household for the analysis of housing exclusion:

(1) Living in insecure housing is considered when one or more of the following three variables are present in the household:
  - Precarious tenancy (provided free of charge by other persons or institutions, sublet, illegally occupied).
  - Having suffered a threat (immediate or otherwise) of expulsion from the dwelling due to economic problems in the household.
  - Households in which some member has suffered in the last year or currently suffers physical or psychological mistreatment.
(2) On the other hand, living in inadequate housing appears when the household shows at least one of the following variables:
  - Households in an infra-housing situation (shanty, shack, prefabricated, or similar).
  - Households in dwellings with serious deficiencies in construction, ruin, etc.
  - Households in a situation of severe overcrowding (<15 m^2^/person).
  - Households living in highly degraded environments.
  - Households that do not have basic supplies or equipment (running water, hot water, electricity, sewage disposal).

For the remainder of the paper, housing exclusion is defined as living in insecure housing or inadequate housing.

### 2.1. Variables

Since the aim of the analysis was to measure the impact of some variables on health status, the self-rated health status was taken as the dependent or explained variable. SRH was derived from the self-perception of health in the EINSFOESSA survey using five categories ranging from “very poor health” to “very good health”. For this analysis, these were recoded into three categories: poor (original “very poor” and “poor), regular, and good (original “very good” and “good”), with “good” being the reference category.

Different groups of variables were identified: those related to personal characteristics (gender, age, educational level, and overall life satisfaction), others related to health variables (diagnosed long-term illness, experiencing poverty related hunger/food insecurity, and being uninsured), and aspects of social support (relating to disability, disabled person in household, divorced/relationship breakdown, widow/bereavement (men and women), giving care, and household size).

Additionally, age and household size, overall life satisfaction, and education were considered metric variables. EINSFOESSA uses five classifications of overall life satisfaction from “very unhappy” to “very happy” and nine possible values for education, from “no studies” to “university degree”. Therefore, higher numerical values in both variables correspond, respectively, to higher educational attainment and overall life satisfaction and vice versa. The remaining variables are all binary, with 1 if the individual is female, widowed/bereaved (including men, widowers, women, and widows), divorced (relationship breakdown), or given care (helping or having helped when others have problems), and if at least one household member is a disabled person, has a diagnosed long-term illness, or is experiencing poverty-related hunger.

### 2.2. Statistical Analysis

Due to the characteristics of the dependent variable (a categorical variable with three options), a logistic regression model or multinomial logit was used. This model takes one of the categories as a basis—in this case, category 1 or “good health”—and estimates the probability of belonging to one of the alternative categories—“regular health” or “poor health”—instead of declaring a good health status, as well as the effect of various explanatory or exogenous variables on that differential probability. In other words, the impact of each variable on whether the state of health is good or not is presented, considering two different alternatives. The ratio of the probability of choosing one outcome category over the probability of choosing the baseline category is modeled as a function that depends on a set of explanatory factors, collected in the vector *x*, as well as the impact that the factors have on the probability expressed in the parameter β. Therefore, the coefficients in Table 1 should be interpreted as a greater probability of presenting a regular or bad health status than a good one if they are positive, and as a lower probability in the opposite case. The analysis was performed using the statistical software Stata v.15 for OSX (StataCorp LLC, College Station, Texas, USA).

## 3. Results

The results in Table 1 describe the situation of households in a situation of residential exclusion, whose household heads were generally men—although one-third are women—in middle age, close to 50 years old. With respect to the explained variable, almost two-thirds of respondents reported a perceived good health status and about 12% reported a poor health status. An important finding is the high percentage of people who declared helping others, along with the relevant health coverage, mainly public, of the Spanish society. With regard to households, they were not very large, with three members on average, and the percentage of households that reported suffering or having suffered from hunger, an extreme form of poverty and exclusion with a clear impact on health, is striking. Lastly, the low educational level of the population in a situation of residential exclusion, together with the general degree of satisfaction with life, slightly higher than the average, is also striking.

The multinomial logistic regression model estimated the self-perceived state of health as a dependent variable, as well as the relationship between this variable and the factors described in the previous section. Repeated estimates were performed to select the significant variables, successively removing those without any explanatory power. The final outcome was the best possible model shown in Table 2 with a pseudo *R^2^* = 0.3561.

To facilitate understanding of the results, the estimates of regular and poor health status relative to the reference category (good) are shown in parallel columns. The respective 95% confidence intervals are shown in brackets.

### 3.1. Personal Variables and SRH

The results reported in Table 2 show for these variables the signs expected a priori, as well as slight differences in significance for both categories of the dependent variable. Starting with the personal variables most related to sociodemographic issues, gender—being a woman—increased the possibility of having a poor state of health, although this effect ceased to be significant when the individual’s state of health was regular. Age, as stated in most previous research, increased the risk of worsening health or, at least, of participants personally evaluating their state of health as poorer. This effect was significant at 5% for both categories of perceived health status. In both cases, it was estimated that an older person had a greater probability of having a worse health status, although there were no significant differences between regular and poor health status. Educational attainment showed the opposite effect to age as expected, again being a significant variable for both levels of health. According to the results of Table 2, a higher level of education improved the perceived state of health and this was likely to relate to better nutrition and greater access to preventive health, as derived from the sign of the coefficients. Being negative, they indicated that a higher level of education reduced the probability of reporting a bad or fair health status versus a good one. Moreover, this reduction effect increased as the good health status was compared to worse situations. Lastly, the effect of overall life satisfaction was considered. A priori, a satisfied person in all areas of his life was expected to perceive their health status better. The results confirmed this hypothesis; for both alternative health states, the coefficients were significant at 5% and showed the expected signs.

### 3.2. Health Variables and SRH

While the health variables introduced into the analysis may also be considered “personal”, the aim was to specifically measure the incidence of various health problems in the population surveyed and their relevance to the state of self-perceived health. The most significant variable with the greatest effect was having a diagnosed disease. This variable had a strong risk reducing effect of having a regular state of health, an effect that was doubled for a poor state of health. These results seem to indicate that, despite being ill, knowing this improves the subjective state of health (this apparently inconsistent result is explained in more detail in Section 4). Along with this factor, being or having recently gone hungry was used as a proxy for nutritional conditions. Again, it was a significant variable that, in this case, worsened health status with a greater impact on poor health status. Lastly, although the national health system in Spain is universal, it is possible to find people without health coverage in the socially excluded population. In this case, the variable was only significant for poor health status with a notable effect of increasing the risk of that status.

### 3.3. Social Support and SRH

The third group of variables related to the ability to have personal relationships with others, called social support herein. Specifically, two of the variables indicated whether the person lived with a partner, differentiating the reason: divorce/relationship breakdown, which was a nonsignificant variable for both alternative states of health, and widowhood/partner bereavement, which was significant for regular and poor health states, having the effect of increasing the probability of not perceiving a good state of health. Being widowed further increased the risk for the worst level of health with respect to the intermediate one. A third variable related to relational aspects included the possibility of giving care. According to Table 2, giving care, whether family members or not, worsened the state of health, probably due to the additional workload. However, as the alternative state of health worsened, this effect was lower in magnitude and in significance; it was significant at 5% for the regular state of health and at 10% for the poor state of health.

Among this set of variables related to the household rather than to the person, different phenomena were considered. The first of these was the presence of a person with a disability in the household. This was the factor with the greatest effect on the probabilities of not having a good state of health. It is important to point out that it does not refer to the interviewed person having a disability, but to at least one person in the household being disabled. In this case, the impact was doubled when explaining a poor or very poor state of health compared to a regular state of health. This fact may explain the result obtained for another of the variables, the household size. In this case, a larger size reduced the risk of having a state of health that was different from good, with a significance of 5% in both cases. The probability reducing impact increased when the alternative was a poor health status.

## 4. Discussion

The first goal of this paper was to analyze the effect of some personal variables on SRH. Our results, in line with previous studies [14,25,26,48], demonstrated how being a woman led to higher likelihood of poor health than good health, in comparison to being a man. Being young and having a high educational level were variables that increased the likelihood of good health with respect to regular and poor health. The scientific literature shows different results regarding health and gender. The influence of gender on SRH diverges depending of the country and the date of data collection. In the year 2004, the Survey of Health, Aging, and Retirement in Europe (SHARE) showed differences between gender and SRH, with higher rates of poor health in females; however, when diseases were added to the equation, the odds of females rating their health poorly were insignificant in Belgium, Italy, and Spain. Indeed, as Crimmins et al. (2011) reported for 11 European countries, if men and women had the same disabilities and diseases, men in five countries would report worse health than women, whereas women would report worse health in no countries [85]. Wolf et al. (2016) found a lack of robust evidence on the health of homeless women in Europe [86]. More research is needed in this area. Looking at the educational level, the results obtained herein were similar to those obtained previously with a Spanish sample of the European Union statistics on income and living conditions (EU-SILC) in the year 2014, where the educational level was the most important personal variable in preventing poor health [87]. These results also appeared in the Spanish homeless population [14]; thus, developing education programs could prevent health difficulties. In relation to the age variable, research demonstrated that older homeless people have higher rates of geriatric syndromes than the general older population [88] and are admitted to hospital 10 or 15 years earlier than the general population [89]. Our results showed a similar situation with people experiencing housing exclusion, where advanced age was a risk factor. An important distinction can be made between old people who were previously homeless for a long time and those who first end up homeless at an older age, and research has shown that the life expectancy among people experiencing homelessness is a lot lower [90].

At the same time, our results showed how high life satisfaction is a preventive factor for good health. These results for the Spanish housing exclusion population agree with other international studies in Mexico [56,61], Turkey [60], China [62], and the United States (US) [57]. In this regard, a US population study reported that lower life satisfaction (LS) was inversely related to HRQoL and to the prevalence of adverse health behaviors and chronic disease, leading the authors to conclude that LS may be an important concept for public health research [91].

The second goal of this paper was to analyze the effect of some health variables on SRH. The results showed that having a diagnosed long-term illness was a preventive factor which increased the probability of having good health with respect to regular or poor health in the Spanish housing exclusion population. While this may look like a counterintuitive contradiction, it could also provide motivation for frequent health checks, where greater consciousness of health status encourages greater self-care. To reassure patients of the best possible health outcome from treatment, it is crucial to make the correct diagnosis as soon as possible. Getting the diagnosis right will enable tailored decision-making that will benefit the patient’s recovery [63]. However, our results diverge from other studies on populations that were not socially excluded, where diagnostic labeling could harm perceived health [64]. The following question arises: Are these differences related to the conditions of people experiencing housing exclusion or not? More future studies on this topic are needed. Moreover, experiencing poverty-related hunger/food insecurity increased the likelihood of having regular and poor health. These results correspond with previous international research where food insecurity was associated with a higher prevalence of chronic disease in adulthood [92] and with a poor SRH [93,94], even more so in people with experiences of social exclusion and racial discrimination [95]. In the same direction, being uninsured increased the probability of having regular and poor health. These results agree with international studies that associated being uninsured to poor health [96,97] and mortality [98]. Previous research on Spanish people experiencing homelessness showed how people who had a health card (public insurance) were significantly more likely to perceive good health than poor health [14].

Lastly, the third aim of the paper was to analyze the effect of some variables related to social support on SRH. Our results showed that widowhood/partner bereavement increased the probabilities of poor health. Research suggests that, although the effect of divorce on health tends to be temporary (not significative in our results), the effect of widowhood is more likely to endure [99]. Our results agree with results in the European Union, where, in many cases, being a widow was connected to living alone and this situation was associated with worse health [100] and lower life satisfaction in comparison to people living with a partner [101], while single-person households were also at greater risk of poverty and social exclusion. This is a widespread phenomenon. In England, France, Sweden, and Germany, more than one-third of all households live alone and, in countries such as Canada, Russia, and Spain, more than one-quarter of homes are single-person households [102]. Research suggests that, in order to save or extend lives, it is important that healthcare providers should, during patient examinations, assess isolation [103]. From a health perspective, this is highly relevant; we can expect that, during current circumstances, with a global coronavirus pandemic, there will be an increase in social isolation and involuntary loneliness.

Previous studies have shown marital status to be associated with better physical and psychological wellbeing. The typical focus of analysis in research on marriage and mental health has been internalizing problems (usually depression or symptoms of mental dysfunction) and externalizing problems such as substance use and abuse (especially alcohol) [104,105]. The findings of SEM analysis demonstrated that being married was significantly associated with a higher level of perceived social support for both genders [106]. In prior research, single individuals were found to report higher levels of depression, anxiety, mood disorders, adjustment problems, and other forms of psychological distress, as well as a higher rate of alcohol-related problems [107,108]. In addition, popular social stereotypes that depict single individuals as miserable, lonely, unhappy, insecure, more neurotic, less satisfied with their lives, with lower self-esteem, less satisfied with their relationship status, and desiring to change their relationship status compared to individuals in relationships [109,110] enhance a negative view of single individuals’ mental health.

The data suggest that people who have a responsibility to care for others have worse SRH. Having a disabled person in the household appeared to be a powerful variable that increased the probability of poor health with respect to good health. These results may reflect deficiencies in the care system in Spain, since most of the care for these persons is carried out by family members with a consequent effect on the carer’s personal health [2]. In comparison, household size increased the probability of having good health with respect to poor health. The results can be explained, on the one hand, by the greater capacity for mutual help as more people live in the household and, on the other, by the positive effect on the perception of health of intrafamily relationships. It is important to remember that, when the number of occupants is higher than the number of rooms, it is defined as overcrowding by Eurostat [111]. This is considered as possible inadequate housing (severe overcrowding, <15 m^2^/person); thus, the number of people in the house is only considered a positive factor for health when it is not causing a severe overcrowding situation. That said, social contact has a positive influence on life satisfaction and is simultaneously associated with good SRH [101]. Other similar results showed how living with parents or grandparents increased individual social capital, with healthy people living in two-generation households having longer life expectancy than healthy people living on their own [112].

Limitations and Future Research Directions

It is important to clarify some possible limitations of the research paper. Although we argued for the validity of SRH, the use of self-reporting for general health and the remainder of the associated variables analyzed may be considered itself a limitation. Data are self-reported and subject to recall and social desirability bias. However, this is a common methodology used in big national surveys in Spain [26,27] and traditionally recommended to be used internationally [32,35]. However, there is scope for future research to analyze and compare SRH with objective measures of health in the Spanish housing exclusion population. According to the results, future research directions could be oriented to develop interventions that could increase the factors that promote good health and a system for early warnings [113]. At the same time, it is crucial to further investigate social and health policies that could remove the risk factors that influence the SRH of people in housing exclusion.

## 5. Conclusions

Recognizing that the living environment influences individual and public health, this paper used the ETHOS definition of housing exclusion and EINSFOESSA survey of social exclusion in Spain to examine the influence of key psychosocial variables on the health of people living in situations of housing exclusion. While housing is an important determinant of health and substandard housing is a major public health issue, there has been no prior analysis of influences on the health of the population living in housing exclusion. The impact of inadequate housing on health in Spain is among the highest in the European Union, but there are no rigorous data on the specific risk and preventive factors that influence the SRH of people experiencing housing exclusion. The new analysis presented here revealed the influence of personal characteristics, health variables, and social support on the SRH of people experiencing housing exclusion in Spain.

The logistic regression model identified risk and prevention factors to inform interventions to improve the health of those living in housing exclusion. The statistical analysis revealed several patterns across the range of psychosocial variables considered for households living in situations of insecure and inadequate housing. Being female was associated with poorer health, as was the process of aging. Higher levels of education were associated with better health (and lower education levels with poorer health). Having a diagnosed long-term illness may be a preventive factor against poorer health, where the knowledge of the illness precipitates regular health interventions and results in individuals taking better care of their health. Effects of poverty such as experiencing poverty-related hunger/food insecurity increased the probability of regular and bad health against good health. As with other societies which have a public health system, those who were not insured for that system faced an increased probability of poor health. Among social variables, the experience of widowhood/bereavement was associated with increased reporting of regular or poor health (rather than good health). Having caring responsibilities for others increased the likelihood of reporting only regular or good health, and this was also the case where a member of the household was disabled. While our findings indicate associations rather than explain causal effects, they nonetheless emerge from a robust dataset and rigorous analysis. Overall, the analysis would support the following possible health and welfare interventions to improve the health status of those living in housing exclusion:

(1) Targeted health interventions toward women and older age groups;
(2) Targeted interventions toward households who have experienced bereavement to tackle both social isolation and economic disadvantages faced by single-person households;
(3) Targeted support toward those with caring interventions and disabled people;
(4) Strategies to optimize educational achievement;
(5) Approaches to maximize health insurance coverage and ensure that existing health conditions are diagnosed and effectively treated, encouraging improved personal healthcare;
(6) Measures to reduce poverty and ensure improved food security.

As the situation of housing exclusion was a “given” factor in the analysis, the results do not address the fundamental question of tackling housing exclusion itself as a social problem. However, a very substantial evidence base exists on the parallel benefits of improved housing, as well as appropriate healthcare and social support, in improving the health and wellbeing of disadvantaged citizens [114,115].

## Figures and Tables

**Table 1:** Descriptive statistics.

Variable	*N*	Percentage
Male household head	949	60.34%
Female household head	624	39.66%
Divorced/relationship breakdown	259	15.82%
Widow/bereavement	107	6.79%
Giving care	1129	71.73%
Disabled person in household	255	16.20%
Diagnosed long-term illness	392	24.90%
Experiencing poverty related hunger/food insecurity	198	12.58%
Uninsured	18	1.14%
**Variable**	**Mean**	**SD**
Age	48.34	0.4066
Educational level	3.02	0.0275
Overall life satisfaction	3.03	0.0292
Household size	3.38	0.4768
**Explained Variable**	***N***	**Percentage**
Good health	1049	66.64%
Regular health	338	21.44%
Bad health	170	11.91%

Source: FOESSA Survey of Social Integration and Needs (EINSFOESSA) 2018.

**Table 2:** Estimation of health status (“good” status fixed as base outcome).

Psychosocial Variables	Variables	“Regular” vs. “Good”	“Poor” vs. “Good”
Personal characteristics	Gender	0.1363 (−0.2332; 0.5057)	0.5319 ** (0.0063; 0.0444)
	Age	0.0269 ** (0.0151; 0.0386)	0.0298 ** (0.0123; 0.0473)
	Educational level	−0.2207 ** (−0.3936; −0.0478)	−0.3945 ** (−0.6553; −0.1360)
	Overall life satisfaction	−0.3332 ** (−0.4869; −0.1795)	−0.5697 ** (−0.7960; −0.3436)
Health characteristics	Diagnosed long-term illness	−2.6551 ** (−3.0570; −2.2532)	−4.4731 ** (−5.0740; −3.8723)
	Experiencing poverty related hunger/food insecurity	0.4015 * (−0.0687; 0.8718)	0.8427 ** (0.1859; 1.4994)
	Uninsured	0.4257 (−1.1615; −2.0128)	2.2749 ** (0.5124; 4.0374)
Social Support	Divorced/relationship breakdown	0.2733 (−0.1888; 0.7354)	0.3058 (−0.3218; 0.9335)
	Widow/bereavement	0.8009 ** (0.0921; 1.5098)	0.8246 * (−0.0872; 1.7365)
	Giving care	0.6127 ** (0.2304; 0.9950)	0.4986 * (−0.0441; 1.0414)
	Disabled person in household	0.5958 ** (0.1327; 1.0590)	1.3735 ** (0.8273; 1.9198)
	Household size	−0.1648 ** (−0.2660; −0.0636)	−0.1067 (−0.2577; 0.0444)
	Constant	3.7087 ** (2.3728; 5.0446)	5.7211 ** (3.8264; 7.6157)
	Pseudo *R^2^*	0.3561	
	Sample size	1574	

Source: Authors’ elaboration from Stata 15. ** 5% significance, * 10% significance.
